# Supplementary material for: Mycoplasma genitalium infection and adverse pregnancy outcomes among pregnant women in South Africa: prospective cohort study
Source: medRxiv. 2026 Aug 11:2026.08.09.26360025. Preprint. [Version 1] doi: 10.64898/2026.08.09.26360025 (PMC13479905; doi:10.64898/2026.08.09.26360025)
Supplement: Supplementary file 2 [file media-2.pdf]

Supplementary file 3: supplementary tables and figures

Supplementary file 3: supplementary tables and figures 1

Table S1: Unadjusted analysis of gestational age at birth, according to baseline characteristics 2

Table S2: M. genitalium exposure at 30-34 week follow-up visit and associations with gestational age at birth and other secondary outcomes 3

Table S3 Adjusted estimates of the impact of exposure to *M. genitalium* and vaginal dysbiosis on birth outcomes in all women 4

Table S4: Adjusted estimates of the impact of exposure to *M. genitalium* and vaginal dysbiosis on birth outcomes in women with live births. 5

Table S5: Adjusted estimates of the impact of exposure to *M. genitalium* at 30-34 weeks gestation on birth outcomes in women who attended their 30-34 week follow-up visit 6

Figure S2: Density plots of gestational age at delivery for 560 women, presented separately for each sexually transmitted infection or vaginal dysbiosis 8

Figure S3: Density plots of gestational age at delivery for 524 women who tested positive to each STI (and no other) in comparison to a reference group with no STIs present 9

Figure S4: Density plots of gestational age at delivery for women who did not receive treatment with azithromycin 10

Figure S5: Quantile regression estimates for the effect of *M. genitalium* exposure on gestational age at birth, in weeks, and birthweight, in grams 11

Table S1: **Unadjusted analysis of gestational age at birth, according to baseline characteristics**

**Characteristic**	**Gestational age at birth, in weeks+days**			**p value**
	**Median**	**IQR**	**Min, max**	
All	39+0	37+4, 40+1	4+1, 44+3	
Age				0.01
Younger than 25 years	39+3	38+0, 40+3	8+5, 43+0	
25 to 35 years old	39+0	37+5, 40+1	4+1, 43+6	
35 years or older	38+5	36+2, 39+6	7+3, 44+3	
Educational level				0.83
Less than grade 10	39+2	38+2, 40+0	8+5, 43+6	
Grade 10 or 11	39+0	37+6, 40+1	7+3, 42+6	
Grade 12 or higher	39+1	37+2, 40+2	4+1, 44+3	
Alcohol consumption				0.95
No	39+0	37+3, 40+1	4+1, 44+3	
Yes	39+0	38+0, 40+1	7+6, 43+6	
Living with HIV				0.40
No	39+0	37+4, 40+1	7+3, 43+6	
Yes, on ART	38+6	37+2, 40+1	4+1, 41+6	
Yes, not on ART	39+3	38+3, 40+3	17+1, 44+3	
Syphilis				0.83
No	39+0	37+4, 40+1	4+1, 44+3	
Yes	39+0	37+1, 40+4	8+1, 42+0	
At least one prior preterm birth				0.42
No	39+0	37+4, 40+1	4+1, 43+6	
Yes	38+5	37+2, 39+5	21+0, 44+3	
Vaginal dysbiosis				0.55
Nugent 0-3	39+0	37+2, 40+1	7+3, 44+3	
Nugent 7-10	39+0	37+5, 40+2	4+1, 43+0	
C. trachomatis				0.13
Negative	39+0	37+3, 40+1	4+1, 44+3	
Positive	39+2	38+3, 40+2	7+6, 43+6	
N. gonorrhoeae				0.15
Negative	39+0	37+4, 40+1	4+1, 44+3	
Positive	38+5	36+2, 39+4	13+5, 40+4	
T. vaginalis				0.23
Negative	39+0	37+4, 40+2	7+3, 44+3	
Positive	38+5	37+3, 39+6	4+1, 43+0	
Candida spp.				0.39
Negative	39+1	37+4, 40+2	4+1, 44+3	
Positive	38+6	37+4, 40+0	8+0, 42+2	
Azithromycin treatment for *C. trachomatis*				0.96
No	39+0	37+3, 40+1	4+1, 44+3	
Yes	39+0	37+6, 40+1	7+6, 43+0	

Data are median and interquartile range (IQR); Min=minimum; Max=maximum.

***Table S2: M. genitalium* exposure at 30-34 week follow-up visit and associations with gestational age at birth and other secondary outcomes**

**Birth outcome**	**Total (N = 441[a])**	***M. genitalium* negative (N = 415)**	***M. genitalium* positive (N = 19)**	***M. genitalium* missing (N = 7)**	**p value**
Gestational age, in days					0.7
Median	39+2	39+2	39+4	37+5	
Q1, Q3	38+1, 40+2	38+1, 40+2	38+3, 40+0	33+1, 39+2	
Min, max	26+2, 43+0	30+4, 43+0	37+1, 42+2	26+2, 41+4	
Missing, n (%)	4	4	0	0	
Live birth					1
Yes, n (%)	434 (99%)	408 (99%)	19 (100%)	7 (100%)	
No, n (%)	3 (1%)	3 (1%)	0 (0%)	0 (0%)	
Missing, n	4	4	0	0	
Preterm birth					0.09
No, n (%)	377 (86%)	354 (86%)	19 (100%)	4 (57%)	
Yes, n (%)	60 (14%)	57 (14%)	0 (0%)	3 (43%)	
Missing, n	4	4	0	0	0.85
Birth weight, in grams					
Median	3144	3160	3200	2760	
Q1, Q3	2850, 3500	2850, 3505	2850, 3475	2165, 3035	
Min, max	1080, 4900	1270, 4900	2200, 4110	1080, 3600	
Missing, n (%)	20	20	0	0	
Low birth weight					0.72
No, n (%)	368 (87%)	347 (88%)	16 (84%)	5 (71%)	
Yes, n (%)	53 (13%)	48 (12%)	3 (16%)	2 (29%)	
Missing, n	20	20	0	0	

[a] 441 of 584 women attended their 30-34 week follow-up visit

Q1, first quartile; Q3, third quartile; Min, minimum; Max, maximum.

Table S3 **Adjusted estimates of the impact of exposure to *M. genitalium* and vaginal dysbiosis on birth outcomes in all women**

Outcome	Comparison[a]	Nugent score 0-3	Nugent score 4-10
Gestational age, in weeks+days	*M. genitalium* not detected	38+5 (38+2, 39+2)	39+0 (38+4, 39+3)
	M. genitalium detected	39+5 (38+5, 40+4)	38+3 (37+6, 39+0)
	Median difference	0+6 (-0+2, 2+0)	-0+4 (-1+2, 0+1)
Miscarriage or stillbirth	Marginal odds ratio	1.74 (0.39, 7.72)	1.74 (0.39, 7.72)
	Risk difference	0.02 (-0.04, 0.08)	0.02 (-0.05, 0.09)
Preterm birth	Marginal odds ratio	1.04 (0.35, 3.12)	1.04 (0.35, 3.12)
	Risk difference	0.01 (-0.16, 0.17)	0.01 (-0.14, 0.15)
Birth weight, in grams	*M. genitalium* not detected	3018 (2821, 3215)	3099 (2912, 3286)
	M. genitalium detected	3116 (2610, 3623)	3002 (2725, 3279)
	Median difference	98 (-543, 739)	-97 (-430, 236)
Low birth weight	Marginal odds ratio	0.95 (0.25, 3.59)	0.95 (0.25, 3.59)
	Risk difference	-0.01 (-0.19, 0.18)	-0.01 (-0.15, 0.14)

[a] For gestational age, comparisons are marginal medians and median differences. For other birth outcomes, comparisons are marginal odds ratios and risk differences. Estimates are adjusted for: age, education, alcohol consumption at baseline, previous preterm births, living with HIV, receipt of azithromycin, and exposure to *C. trachomatis, T. vaginalis, N. gonorrhoeae* or *Candida spp*. Quantile regression models include an interaction term between *M. genitalium* and vaginal dysbiosis.

Table S4: **Adjusted estimates of the impact of exposure to *M. genitalium* and vaginal dysbiosis on birth outcomes in women with live births.**

**Outcome**[a]	**Comparison**[b]	**Nugent score 0-3**	**Nugent score 4-10**
Gestational age, in weeks+days	*M. genitalium* not detected	38+5 (38+1, 39+2)	39+0 (38+4, 39+3)
	M. genitalium detected	39+4 (39+0, 40+1)	38+4 (38+0, 39+1)
	Median difference	0+6 (0+1, 1+3)	-0+3 (-1+1, 0+1)
Preterm birth	Marginal odds ratio	0.75 (0.18, 3.23)	0.75 (0.18, 3.23)
	Risk difference	-0.04 (-0.21, 0.14)	-0.03 (-0.17, 0.11)
Birth weight, in grams	*M. genitalium* not detected	3010 (2818, 3203)	3093 (2910, 3275)
	M. genitalium detected	3110 (2626, 3594)	2993 (2710, 3277)
	Median difference	100 (-512, 711)	-99 (-442, 244)
Low birth weight	Marginal odds ratio	0.98 (0.26, 3.72)	0.98 (0.26, 3.72)
	Risk difference	0 (-0.2, 0.19)	0 (-0.16, 0.15)

[a] Estimates are not shown for miscarriage or stillbirth, as this analysis excluded women with these outcomes.

[b] For gestational age, comparisons are marginal medians and median differences. For other birth outcomes, comparisons are marginal odds ratios and risk differences. Estimates are adjusted for: age, education, alcohol consumption at baseline, previous preterm births, living with HIV, receipt of azithromycin, and positive test results for *C. trachomatis*, *T. vaginalis, N. gonorrhoeae* or *Candida spp*. Quantile regression models include an interaction term between *M. genitalium* and vaginal dysbiosis.

Table S5: **Adjusted estimates of the impact of exposure to *M. genitalium* at 30-34 weeks gestation on birth outcomes in women who attended their 30-34 week follow-up visit**

**Outcome**[a]	**Comparison**[b]	**Estimate**
Gestational age, in weeks+days	*M. genitalium* not detected	39+1 (38+5, 39+5)
	M. genitalium detected	39+2 (38+4, 40+0)
	Median difference	0+1 (-0+4, 0+6)
Birth weight, in grams	*M. genitalium* not detected	3135.51 (2874.98, 3396.03)
	M. genitalium detected	2965.33 (2536.34, 3394.31)
	Median difference	-170.18 (-542.65, 202.29)
Low birth weight	Marginal odds ratio	1.57 (0.41, 6.03)
	Risk difference	0 (-0.02, 0.02)

[a] 143 women who did not attend their 30-34 week follow-up visit are excluded. Since most occurrences of miscarriage, stillbirth or preterm birth occurred before this visit, we do not show estimates for these outcomes and do not present results among women with live births. Analyses are performed on complete cases, excluding women with missing data.

[b] For gestational age, comparisons are marginal medians and median differences. For low birthweight, comparisons are marginal odds ratios and risk differences. Estimates are adjusted for: age, education, alcohol consumption at baseline, previous preterm births, living with HIV, receipt of azithromycin, and exposure to vaginal dysbiosis, *C. trachomatis*, *T. vaginalis*, *N. gonorrhoeae* or *Candida spp.* at follow-up.

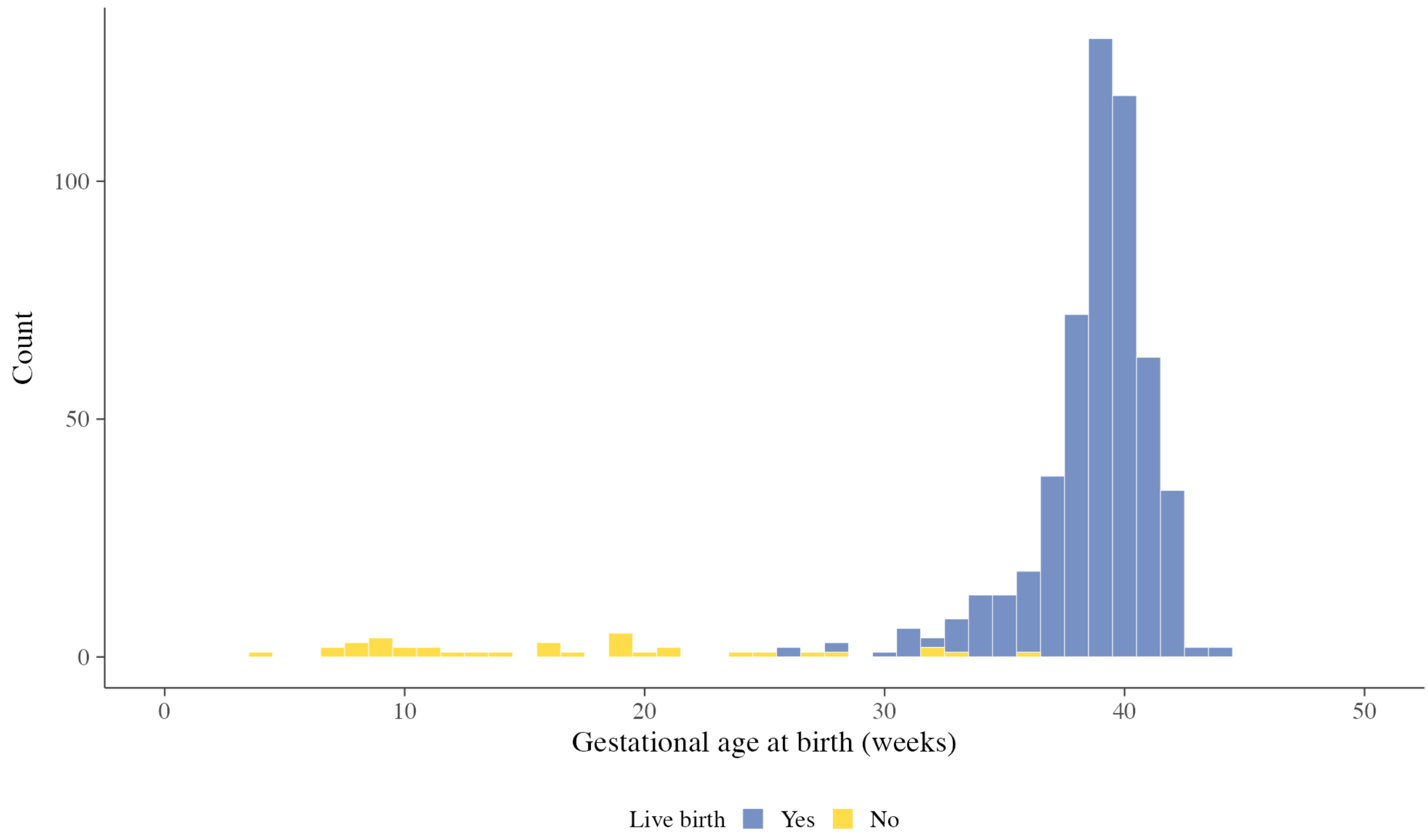

***Figure S1*: Histogram of gestational age at delivery for 584 women**

Blue bars indicate women with live births, yellow indicates stillbirth, miscarriage or neonatal death

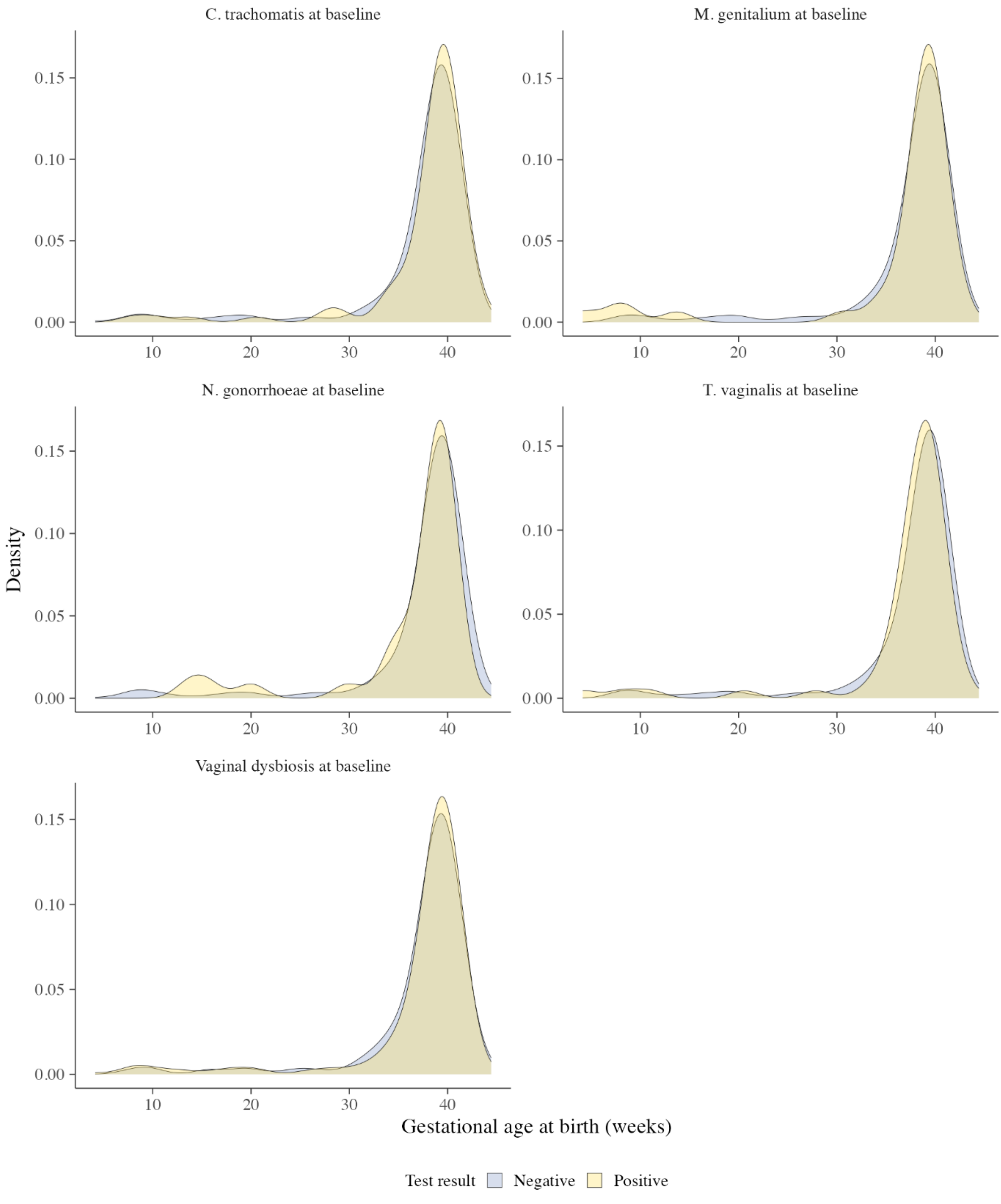

Figure S2**: Density plots of gestational age at delivery for 560 women, presented separately for each sexually transmitted infection or vaginal dysbiosis**

Blue indicates densities for women who tested negative to the infection, yellow indicates densities for women who tested positive. Densities were estimated with a Gaussian kernel and bandwidth of 1.5.

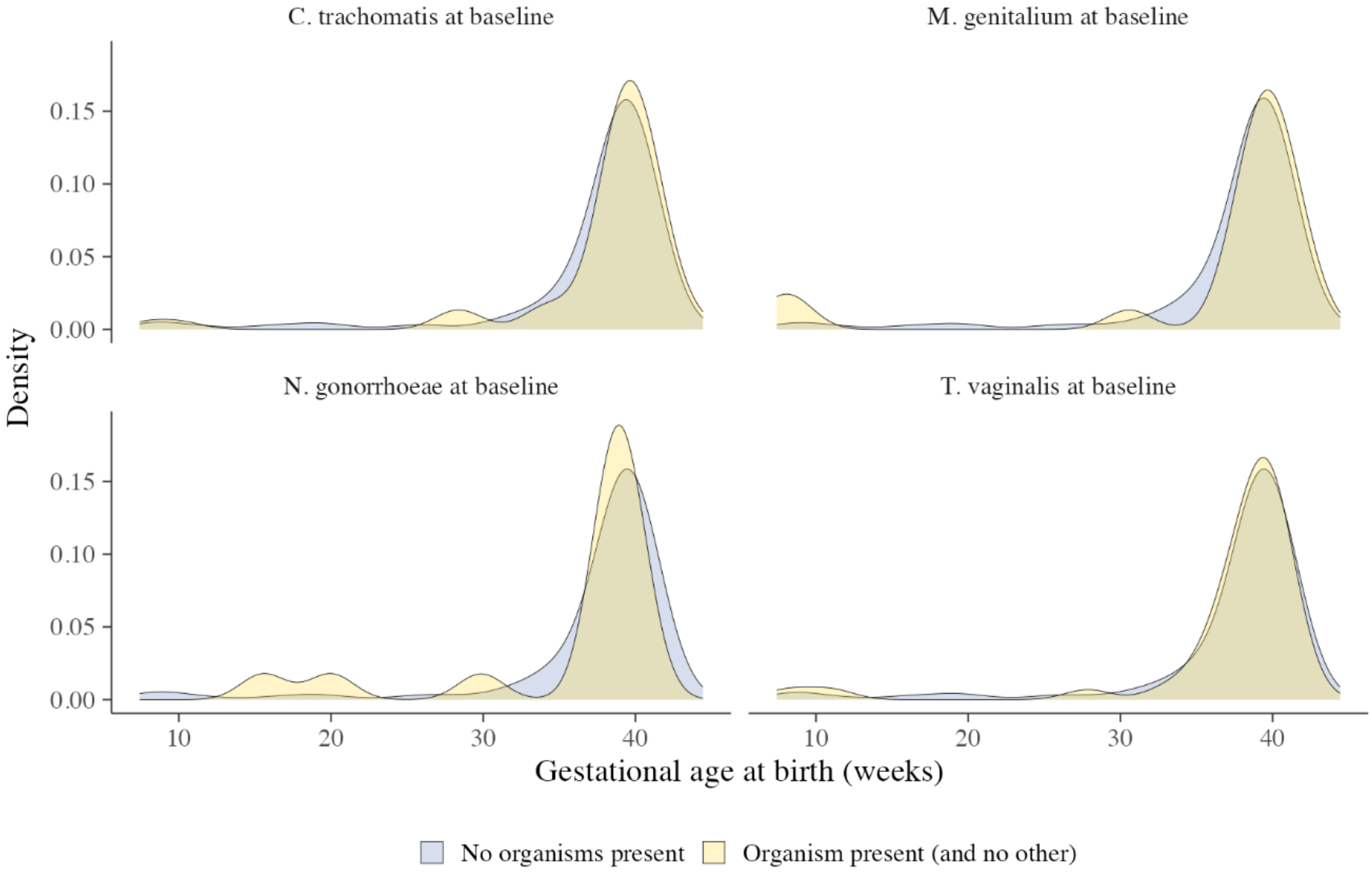

**Count**	***C. trachomatis***	***M. genitalium***	***N. gonorrhoeae***	***T. vaginalis***
No organisms present	466	504	509	485
Organism present (and no other)	58	20	15	39

***Figure S3*: Density plots of gestational age at delivery for 524 women who tested positive to each STI (and no other) in comparison to a reference group with no STIs present**

The reference group with no organisms present, shown in blue, includes women who tested negative for all of the following organisms at baseline: *M. genitalium, T. vaginalis, C. trachomatis, N. gonorrhoeae*. The comparison group, shown in yellow, includes women who tested positive at baseline for only the given STI, and no other. Women with missing test results or gestational age at delivery are excluded. Densities were estimated with a Gaussian kernel and bandwidth of 1.5.

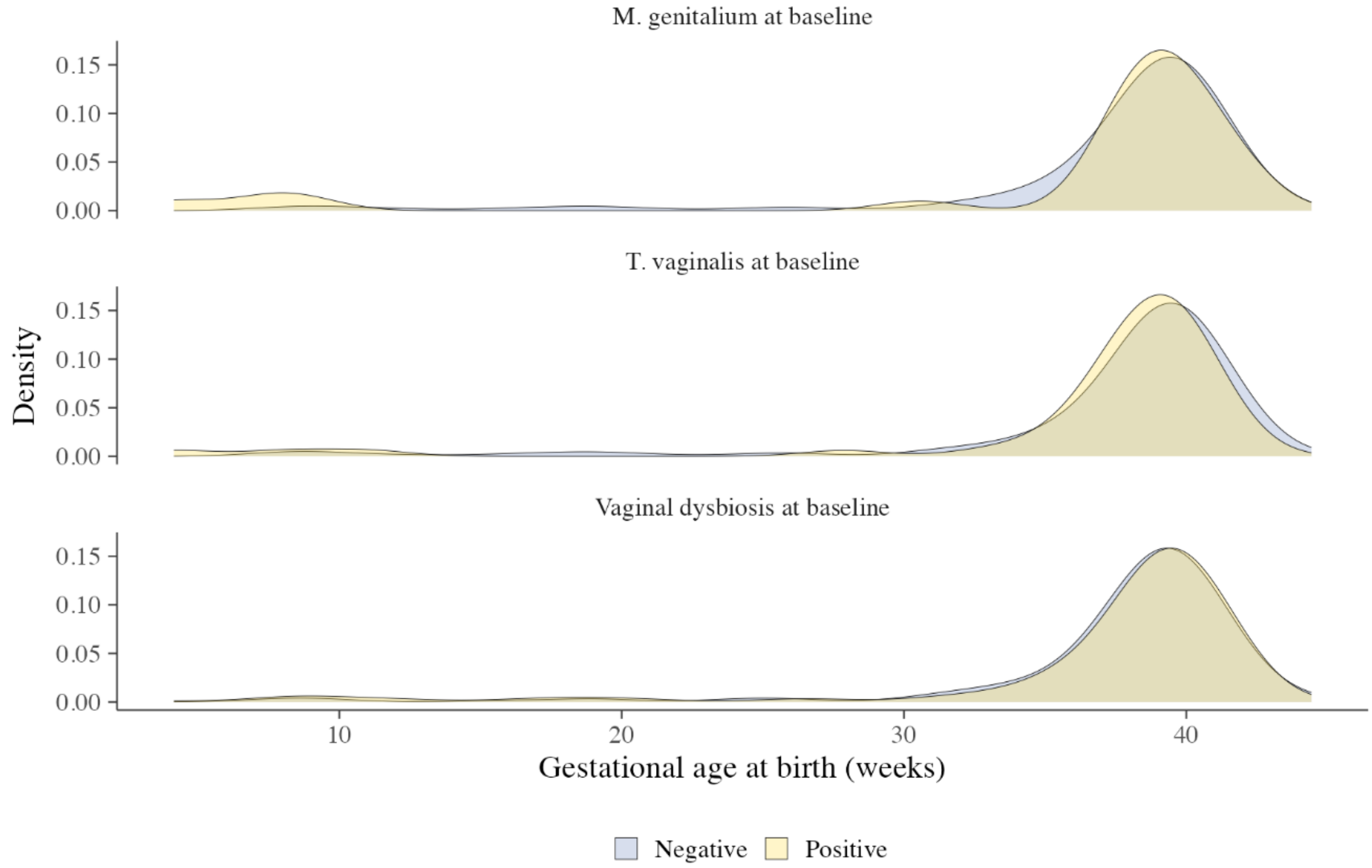

	M. genitalium	*T. vaginalis*	Vaginal dysbiosis
Organism not present	413	396	169
Organism present	27	44	271

***Figure S4*: Density plots of gestational age at delivery for women who did not receive treatment with azithromycin**

Women with missing test results or gestational age at birth are excluded, along with women who received treatment with azithromycin. Density plots for *C. trachomatis* and *N. gonorrhoeae* are not shown due to small counts, as almost all women who tested positive to these two organisms at baseline received treatment. Blue indicates densities for women who tested negative to the infection, yellow indicates densities for women who tested positive. Densities were estimated with a Gaussian kernel and bandwidth of 1.5.

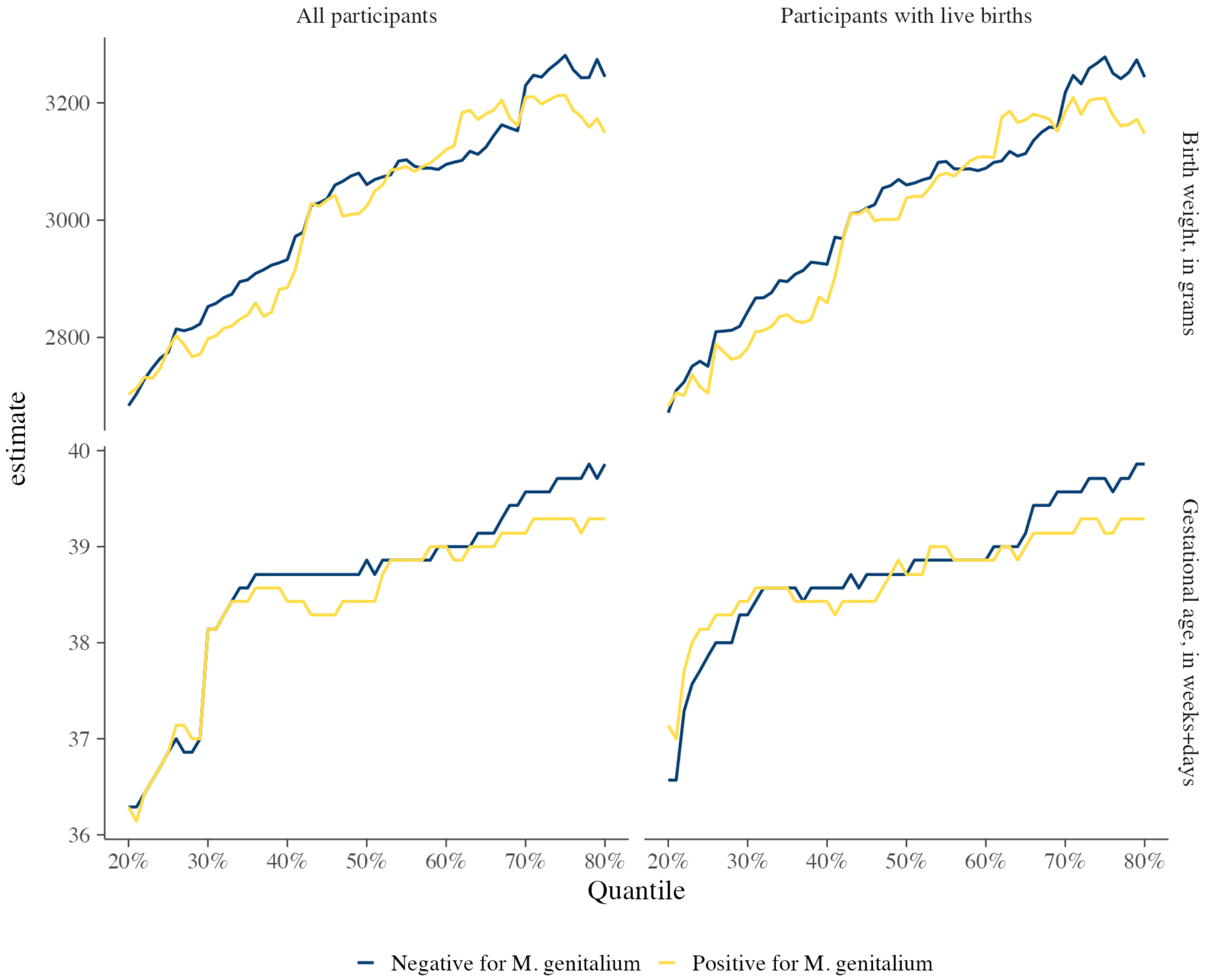

***Figure S5*: Quantile regression estimates for the effect of *M. genitalium* exposure on gestational age at birth, in weeks, and birthweight, in grams**

Lines indicate marginal estimates of gestational age at birth or birth weight for each given quantile, shown separately for women with and without *M. genitalium*. Results are shown for regression estimates fitted to all women (left) and only to women with live births (right).
