# Supplementary material for: Mycoplasma genitalium infection and adverse pregnancy outcomes among pregnant women in South Africa: prospective cohort study
Source: medRxiv. 2026 Aug 11:2026.08.09.26360025. Preprint. [Version 1] doi: 10.64898/2026.08.09.26360025 (PMC13479905; doi:10.64898/2026.08.09.26360025)
Supplement: Supplementary file 3 [file media-3.pdf]

Supplementary material 1: STROBE checklist for cohort studies

Item		Item	Recommendation	Page, section
Title and abstract		1	(a) Indicate the study's design with a commonly used term in the title or the abstract	1
			(b) Provide in the abstract an informative and balanced summary of what was done and what was found	2
Introduction				
	Background/rationale	2	Explain the scientific background and rationale for the investigation being reported	3, Introduction, para 1-3
	Objectives	3	State specific objectives, including any prespecified hypotheses	3, Introduction, para 3
Methods				
	Study design	4	Present key elements of study design early in the paper	3-4, Sudy design
	Setting	5	Describe the setting, locations, and relevant dates, including periods of recruitment, exposure, follow-up, and data collection	4, Study setting and population
	Participants	6	(a) Give the eligibility criteria, and the sources and methods of selection of participants. Describe methods of follow-up	4, Study setting and population; p5, Study procedures and visits
			(b) For matched studies, give matching criteria and number of exposed and unexposed	Not applicable
	Variables	7	Clearly define all outcomes, exposures, predictors, potential confounders, and effect modifiers. Give diagnostic criteria, if applicable	4, Study procedures and visits; 5, Microbiological analyses; 5, Outcomes and statistical analyses
	Data sources/measurement	8	For each variable of interest, give sources of data and details of methods of assessment (measurement). Describe comparability of assessment methods if there is more than one group	4, Study procedures and visits; 5, Microbiological analyses; 5, Outcomes and statistical analyses
	Bias	9	Describe any efforts to address potential sources of bias	3, Methods, 6, Outcomes and statistical analyses
	Study size	10	Explain how the study size was arrived at	5, Outcomes and statistical analyses
	Quantitative variables	11	Explain how quantitative variables were handled in the analyses. If applicable, describe which groupings were chosen and why	5-6, Outcomes and statistical analyses
	Statistical methods	12	(a) Describe all statistical methods, including those used to control for confounding	6, Outcomes and statistical analyses
			(b) Describe any methods used to examine subgroups and interactions	6, Outcomes and statistical analyses
			(c) Explain how missing data were addressed	6, Outcomes and statistical analyses
			(d) If applicable, explain how loss to follow-up was addressed	6, Outcomes and statistical analyses
			(e) Describe any sensitivity analyses	6, Outcomes and statistical analyses
Results				
	Participants	13	(a) Report numbers of individuals at each stage of study—eg numbers potentially eligible, examined for eligibility, confirmed eligible, included in the study, completing follow-up, and analysed	6, Results
			(b) Give reasons for non-participation at each stage	7, Results
			(c) Consider use of a flow diagram	7, Results
	Descriptive data	14	(a) Give characteristics of study participants (eg demographic, clinical, social) and information on exposures and potential confounders	Table 1
			(b) Indicate number of participants with missing data for each variable of interest	Table 1
			(c) Summarise follow-up time (eg, average and total amount)	7, Reported totals followed up to birth
	Outcome data	15	Report numbers of outcome events or summary measures over time	Table 2, Table S1
	Main results	16	(a) Give unadjusted estimates and, if applicable, confounder-adjusted estimates and their precision (eg, 95% confidence	9-11, Tables 2 and 3, Table S1-S5, Fig S2-S4

Item		Item	Recommendation	Page, section
			interval). Make clear which confounders were adjusted for and why they were included	
			(b) Report category boundaries when continuous variables were categorized	Table 2
			(c) If relevant, consider translating estimates of relative risk into absolute risk for a meaningful time period	Not applicable
	Other analyses	17	Report other analyses done—eg analyses of subgroups and interactions, and sensitivity analyses	9, 12
Discussion				
	Key results	18	Summarise key results with reference to study objectives	12
	Limitations	19	Discuss limitations of the study, taking into account sources of potential bias or imprecision. Discuss both direction and magnitude of any potential bias	13
	Interpretation	20	Give a cautious overall interpretation of results considering objectives, limitations, multiplicity of analyses, results from similar studies, and other relevant evidence	13-14
	Generalisability	21	Discuss the generalisability (external validity) of the study results	15
Other information				
	Funding	22	Give the source of funding and the role of the funders for the present study and, if applicable, for the original study on which the present article is based	16

Source: https://www.equator-network.org/reporting-guidelines/strobe/
